# Measurement of daily sodium excretion in patients with chronic kidney disease; special reference to the difference between the amount measured from 24 h collected urine sample and the estimated amount from a spot urine

**DOI:** 10.1080/0886022X.2018.1456452

**Published:** 2018-04-05

**Authors:** Hoichi Amano, Seiji Kobayashi, Hiroyuki Terawaki, Makoto Ogura, Yoshindo Kawaguchi, Takashi Yokoo

**Affiliations:** a Division of Nephrology and Hypertension, Department of Internal Medicine, The Jikei University School of Medicine, Tokyo, Japan;; b Graduate School of Public Health, Teikyo University, Tokyo, Japan;; c Department of Allergy and Rheumatology, Nippon Medical School Graduate School of Medicine, Tokyo, Japan;; d Department of Internal Medicine, Nephrology Teikyo University School of Medicine Teikyo University Chiba Medical Center, Ichihara, Japan

**Keywords:** CKD, salt intake, sodium excretion, a spot urine sample, 24-hour collected urine sample

## Abstract

It is important to grasp a patient’s daily sodium intake in the management of chronic kidney disease, as sodium intake is widely recommended at 6 g/day or less. There are multiple equations widely known for estimating the daily sodium excretion from a spot urine sample, but these are aimed at healthy people. There are few reports that validate equations in patients with chronic kidney disease. The purpose of this study is to evaluate whether the amount of measured daily sodium excretion from a sample collected for 24-h urine (24HU) is equal to that of using an equation from a spot urine sample (SU) in patients with chronic kidney disease. One hundred sixty-two patients with chronic kidney disease from Kanagawa Prefecture Shiomidai Hospital, Japan and the Jikei University Kashiwa Hospital, Japan participated in the study. Daily sodium excretion was measured from 24HU and compared with it from SU by using the formula according to Tanaka et al. Sodium excretion by 24HU was 2744 mg/day and estimating daily sodium excretion from SU was 3315 mg/day. The coefficient of determination was 0.17 (*p* < .001) in multivariate regression analysis. The coefficient of determination was extremely low. Thus, there is a considerable difference between the amount of sodium excretion calculated from a 24HU and that from a SU in patients with chronic kidney disease.

## Introduction

According to the World Health Organization (WHO), the fourth highest risk factor for death due to non-communicable diseases is high sodium intake. The increase of salt intake is associated with hypertension, especially in populations burdened with primary hypertension and the elderly [1,2]. Moreover, high salt intake is associated with higher risk for stroke, cardiovascular diseases, and faster progression to end stage kidney disease [3,4]. In patients with chronic kidney disease (CKD), the event of cardiovascular disease is related to quantity of urinary sodium excretion [5]. Reduction of salt intake promotes lowering blood pressure and proteinuria. This is considerably more effective than the concomitant use of angiotensin receptor blocker (ARB) for control of proteinuria and blood pressure in patient using ACE inhibitor (ACEi) [6]. It was estimated that reducing dietary salt intake by 3 g per day could result in reducing the number of the death from some causes of cardiovascular diseases by 44 000–92 000 per year and as for the medical expenses it may save 10–24 billion dollars per year in the US [7]. The WHO recommends a salt intake of 5 g or less per day for the general population to prevent incidence of cardiovascular diseases [8].

Currently, the daily amount of salt intake recommended for patients with CKD is 6 g or less per day in Japan [9]. However, there is variety of diets with high sodium around the world. The INTERSALT study as a worldwide epidemiologic study of large sample size showed the mean daily salt intake was 9.2 g/day at 52 areas and in 32 countries [10]. In Japan the mean daily salt intake was 11.3 g/day by men and 9.6 g/day by women, respectively [11]. It is crucial to quantify the daily amount of sodium intake in an individual patient, estimated from sodium excretion into urine in clinical practice. Some equations of the daily sodium excretion from a spot urine sample (SU) were proposed, however, these equations have been applied for healthy people only. There have been few reports that have validated the equations in patients with CKD. The purpose of this study is to examine whether daily amount of sodium excretion calculated form a SU approximates that measured from a 24-h urine (24HU) in patients with CKD.

## Materials and methods

### Patient population

We enrolled 162 outpatients with CKD from Kanagawa Prefecture Shiomidai Hospital, Japan and the Jikei University Kashiwa Hospital, Japan from April 2013 to June 2015. Diuretic users were excluded from this study.

#### Data collection

Estimated glomerular filtration rate (eGFR) is expressed by the following formula: eGFR (mL/min/1.73 m^2^) = 194 × creatinine (Cr) − 1.094 age^−0.287^ (× 0.739, if female) prepared from Japanese Society of Nephrology [9]. All participants were educated about the use of the device for proportionally collectable urine at rate of 1/50 (Urine-MateP^R^, Sumitomo Bekelite, Tokyo, Japan) for each voiding during 24-h. Casual SU was obtained at the time visiting outpatient clinic in the morning of the same day when a patient brought 24HU prior to the visit. Daily sodium excretion was calculated from SU by using the formula of Tanaka et al. [12]. The principle of Tanaka’s formula is to estimate the 24-h-sodium excretion by measuring the sodium/Cr ratio from a SU [13]. This formula was generated based on data from healthy volunteers. Tanaka’s formula is recommended for use applying in the estimation of salt intake by the Japanese Society of Hypertension [13]. The formula used in the study was as follow: Tanaka’s formula 24-h-urinary sodium excretion (mmol/day) = 21.98 × urinary sodium/urinary Cr × {−2.04 × age +14.89 × weight (kg) + 16.14 × height (cm) − 2244.45}^0.392^. Urinary concentration of sodium and Cr were measured by Japan Electron Optics Laboratory autoanalyzer, Tokyo, Japan. Sodium (mmol/L) was converted to sodium (mg/L) by using following formula: 1 mmol/L = 23 mg/L.

### Ethical committee

The protocol of the study was approved by the ethical committee of Kanagawa Prefectural Shiomidai Hospital (Approval number 2706) and the ethical committee of the Jikei University Kashiwa Hospital (Approval number 7058). Informed consent was not obtained from an individual patient, because his/her laboratory data used in this study was extracted from the file of routine examinations and were analyzed, retrospectively.

### Statistical methods

Data were expressed as mean ± standard deviation (SD). The correlation between an estimated and a measured sodium excretion was identified by Pearson's correlation coefficients. We used a multivariate regression analysis to assess the measured 24-h-sodium excretion after adjustment for age, sex, eGFR, body mass index (BMI), estimated 24 h urine sodium excretion, diabetes, hypertension, and ACEi/ARB use with various models. A difference with *p* < .05 was considered to be statistically significant. In general, when the obtained regression coefficient is 0.7–1.0, there is a strong correlation. When it is 0.4–0.7, there is slight correlation. When it is 0.2–0.4, there is weak correlation and when it is 0–0.2, there is almost no correlation. All statistical analyses were performed with EZR (Version 1.33, Saitama Medical Center, Jichi Medical University, Saitama, Japan), which is a graphical user interface for R (The R Foundation for Statistical Computing, Vienna, Austria). More precisely, it is a modified version of R commander designed to add statistical functions frequently used in biostatistics.

## Results

### Baseline patient characteristics

The characteristics of the patients are shown in Table 1. Mean age was 64.0 ± 14.3 years and 51.2% of the patients were male. Mean serum Cr was 1.39 ± 1.31 mg/dL and eGFR was 52.8 ± 23.9 mL/min/1.73m^2^. 60.9% of the patients had hypertension,18.5% had diabetes mellitus, and ACEi/ARB were used in 86 patients (53.1%). The urinary Cr excretion in 24HU was 980 ± 321 mg/day. Daily sodium excretion calculated from 24HU was 2744 ± 1330 (mean ± SD) mg/day, while estimated daily sodium excretion from SU was 3315 ± 996 (mean ± SD) mg/day.

**Table 1.** Patient characteristics (*n* = 162)^a^.

Age (years)	64.0 ± 14.3
Male (%)	83 (51.2)
BMI (kg/m^2^)	23.3 ± 3.9
SBP (mmHg)	127.4 ± 16.6
DBP (mmHg)	73.9 ± 12.1
Serum creatinine (mg/dl)	1.39 ± 1.31
eGFR (mL/min/1.73m^2^)	52.8 ± 23.9
Hypertension (%)	98 (60.5)
Diabetes (%)	30 (18.5)
ACEi/ARB use (%)	86 (53.1)
24 h urine volume (mL/day)	1988 ± 737
24 h urine creatinine excretion (mg/day)	980 ± 321
24 h urine sodium excretion (mg/day)	2744 ± 1330
Estimated 24 h urine sodium excretion (mg/day)	3315 ± 996

BMI: body mass index; SBP: systolic blood pressure; DBP: diastolic blood pressure; eGFR: estimated glomerular filtration rate; ACEi: ACE inhibitor; ARB: angiotensin receptor blocker.

a Data are expressed as mean ± standard deviation or numbers with %.

### Analysis result

**Figure 1.**
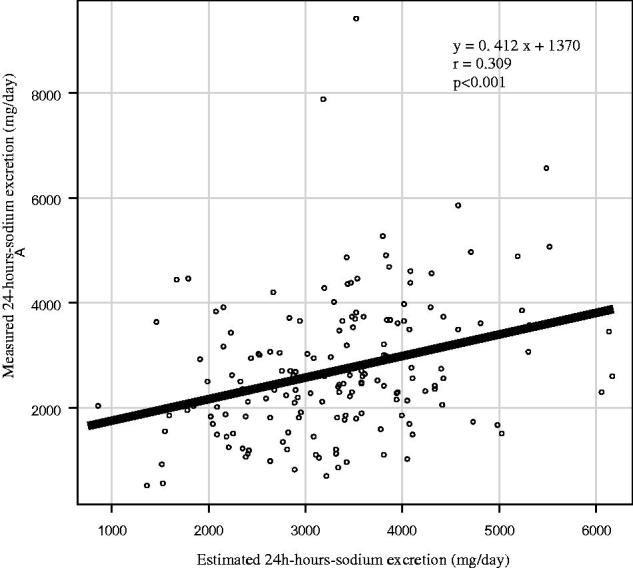
Correlation between measured 24 h-sodium excretion from a 24 h urine sample and estimated 24 h-sodium excretion from a spot urine sample. Estimated regression coefficient was 0.309 (*p* < .001). There is a weak correlation with two methods in regression analysis. *r* indicates the regression coefficient.

The measured 24-h-sodium excretion was correlated with the estimated sodium excretion by SU (Figure 1). Estimated regression coefficient was 0.309 (*p* < .001, 95%CI: 0.162–0.442). The measured 24-h-sodium excretion stratified by eGFR was correlated with the estimated sodium excretion by SU (Table 2). Estimated regression coefficient was 0.407 (*p* < .001, 95%CI: 0.193–0.5830) with eGFR ≧ 60 mL/min/1.73m^2^, 0.216 (*p* = .1, 95%CI: −0.042–0.447 with 31–59 mL/min/1.73m^2^ and <30 mL/min/1.73m^2^, 0.131 (*p* = .482, 95%CI: −0.234–0.464). There was no correlation in patients with eGFR <60 mL/min/1.73m^2^ in univariate analysis. The result of multivariate regression analysis adjusted with various models was shown in Table 3. The age, sex, HT, and use of ACEi/ARB appeared to be dominant factors (Model 4), but the coefficient of determination was 0.17. The predictive performance of an estimated 24-h-sodium excretion from SU turned out low.

**Table 2.** The correlation between estimated sodium excretion by a spot urine and a measured 24 h sodium excretion stratified by eGFR.

eGFR (mL/min/1.73m^2^)	*n*	*r*	95%CI	*p* value
≧ 60	72	0.407	0.193–0.583	<.001
31–59	59	0.216	−0.042–0.447	.1
≦ 30	31	0.131	−0.234–0.464	.482

eGFR: estimated glomerular filtration rate; r: regression coefficient; CI: confidence interval.

**Table 3.** Predictors for measured 24 h urine sodium secretion from measured values in multivariate regression analysis adjusted with various models^a^.

	Model 1	Model 2	Model 3	Model 4
Age (year)		16.10*	16.69*	15.45*
Male		508.48**	518.06**	497.59*
BMI (kg/m^2^)				−16.54
Estimated 24 h urine sodium excretion (mEq/day)	0.41*	0.45***	0.45***	0.48**
eGFR (mL/min/1.73m^2^)			4.48	0.25
Diabetes				270.24
Hypertension				−515.04*
ACEi/ARB use				216.96*
Adjusted R^2^ for models	0.09	0.15	0.15	0.17

BMI: body mass index; eGFR: estimated glomerular filtration rate; ACEi: ACE inhibitor; ARB: angiotensin receptor blocker; R^2^: coefficient of determination.

a Standardized regression coefficients are shown.

* *p* < .05.

** *p* < .01.

*** *p* < .001.

## Discussion

The purpose of this study was to evaluate the difference for daily sodium excretion between the amount obtained from 24HU and that from casual SU in the morning in patients with CKD. Estimated daily sodium excretion obtained by Tanaka’s formula in patients with CKD showed almost no correlation with the amount of measured 24-h-sodium excretion in multivariate regression analysis. The result of this study showed that it was hard to evaluate daily sodium excretion from SU in patients with CKD.

Sodium excretion exhibits a circadian rhythm. In the healthy population, sodium excretion during nighttime has been reported to be 20% lower than that during daytime [14,15]. On the other hand, in patients with CKD, the pattern of sodium excretion differs from the healthy population. When kidney function decreases, it may lead to salt-sensitive hypertension and non-dipper type hypertension [16]. Moreover, salt-sensitive hypertension causes non-dipper type hypertension [17,18]. It is demonstrated that salt-sensitive hypertension and non-dipper type hypertension raise the quantity of sodium excretion during nighttime [19]. According to recent studies, the circadian clock mechanism of the kidney has an important role in controlling the amount of urinary sodium excretion [20,21]. Disorders of rest-activity and feeding cycle cause failure in the renal circadian clock, which causes further urinary sodium excretion rhythm disorder [22]. Considering those explanations, the pattern of sodium excretion in patients with CKD may differ from that of the healthy population. As shown in our results, it is hard to evaluate daily sodium excretion from casual SU.

In this study, the significant findings are that there is a difference in the calculation of the amount of daily sodium excretion between 24HU and SU in CKD patients and the amount of sodium excretion obtained from 24HU is lower than that from SU. There possible reasons why a discrepancy is present in the amount of sodium excretion between 24HU and SU were considered. First, the pattern of sodium excretion in patients with CKD differs from the healthy population; consequently, the estimated daily sodium excretion from SU may become overestimated. Second, generally, urinary sodium excretion falls in accordance with decreasing kidney function. As shown in our study, the correlation between amount of sodium excretion obtained from 24HU and from SU decreases as the kidney function decreases. However, pattern of sodium excretion range widened by person to person. Third, incomplete voiding capability in patients with diabetic neuropathy and prostatic hyperplasia in elderly subjects might affect the feasibility of complete collecting urine for 24 h. Those might cause the lower amount of sodium excretion measured by 24HU.

We have compared past studies similar to this study. The study indicated by Kawasaki et al. [23] was conducted with the healthy population using a second morning urine sample . The estimated regression coefficient was 0.728 (*p* < .001) and the correlation with a 24-h-sodium excretion and estimated sodium excretion using a second morning urine samples is strong. Tanaka et al. [12] indicated there was a significant correlation with the amount of sodium excretion measured from 24HU and that estimated sodium excretion using causal SU. The estimated regression coefficient was 0.54 (*p* < .01). The study reported by Rhee et al. [24] also reported a similar result. However, these three studies were conducted in people with normal kidney function. In our study, patients with impaired kidney function showed a weak correlation and the estimated regression coefficient was lower in comparison with the past studies.

There are only a few research results opposites to our result. Imai et al. [25] showed that Tanaka`s equation for estimating sodium excretion from the first morning urine in patients with CKD is enough for use [25]. The difference between the previous report and ours might be caused by sampling occasion only at the first morning urine or not. This is the point to be emphasized in our study. In practice, it is not easy to obtain the first morning urine sample in out-outpatient setting. Ogura et al. [26] reported that a spot urine could be used to estimate sodium excretion in patients even with low eGFR [26]. However, in their study, subjects who used diuretics which affect sodium excretion were not excluded. Our study excluded users of diuretics. The other study concluded that a spot urine sodium test could be applied in an equation to detect 24-h urine sodium excretion among patients with CKD and healthy kidney function [27]. However, there are also some important limitation in this study. First, the participants eGFR was not shown. Considering that one-third of participation had a healthy kidney function, we could not have confirmed whether the result was correct or not, for participation with kidney function decline. In our study, the eGFR at 52.8 mL/min/1.73m^2^, which was in status of CKD. Second, the formula they used was not validated and the reliability have not been confirmed.

There are some limitations in our study. First, the number of participants was small. Second, we could not confirm the exact timing of sampling of SU. Third, there was also the possibility of sampling error when collecting 24-h urine (e.g., having residual urine in bladder and technical inaccuracy of the urine collection). Generally, it is said that 24 h urine Cr excretion is 1 g/day, so the validity of collecting 24-h urine is considered to be reasonable. Last, but not least, we studied the correlation of only one sample both from 24HU and SU.

In conclusion, we demonstrated that there is a significant difference in daily sodium excretion between the amount obtained from a 24HU and that estimated from a SU in patients with CKD. Clinicians should understand there is a considerable difference in the calculation of the amount of daily sodium excretion between 24HU and SU in patients with CKD.

Part of this study was reported in the 59th Annual Meeting of the Japanese Society of Nephrology, 2016, Yokohama.
